# Reattribution of Auditory Hallucinations Throughout Avatar Therapy: A Case Series

**DOI:** 10.3390/reports8030113

**Published:** 2025-07-18

**Authors:** Sabrina Giguère, Mélissa Beaudoin, Laura Dellazizzo, Kingsada Phraxayavong, Stéphane Potvin, Alexandre Dumais

**Affiliations:** 1 Department of Psychiatry and Addictology, University of Montreal, Montreal, QC H3T 1J4, Canada; sabrina.giguere.2@umontreal.ca (S.G.); melissa.beaudoin.1@umontreal.ca (M.B.); laura.dellazizzo@umontreal.ca (L.D.); stephane.potvin@umontreal.ca (S.P.); 2 Research Center, Institute in Mental Health of Montreal, Montreal, QC H1N 3V2, Canada; 3 Services et Recherches Psychiatriques AD, Montreal, QC H1N 3V2, Canada; kingsada.phraxayavong.cemtl@ssss.gouv.qc.ca; 4 Institut National de Psychiatrie Légale Philippe-Pinel, Montreal, QC H1C 1H1, Canada

**Keywords:** treatment-resistant schizophrenia, auditory verbal hallucinations, avatar therapy, virtual reality, case series

## Abstract

**Background and Clinical Significance:** Avatar Therapy (AT) for individuals with treatment-resistant auditory verbal hallucinations (AVHs) in schizophrenia aims to address emotional responses, beliefs about voices, self-perception, and coping strategies. This study focuses on three participants who, during AT, shifted their belief about the origin of their most distressing voice from an external source to a self-generated one. **Case Presentation:** The objective of this study was to explore the evolution of the reattribution of the participants’ most distressing voice to oneself during AT and the patients’ perception of this reattribution. Immersive sessions and semi-structured interviews were transcribed and qualitatively described to provide a session-by-session account of the evolution of each participant’s AVH reattribution to themselves during the course of AT, along with their perceptions of this reattribution. This process led to the recognition that initially perceived as external voices were internally generated thoughts, reflecting how participants viewed themselves. Two participants reported a reduction in AVH severity. All three described positive changes in how they related to their voices and self-perception. Additional improvements were observed in emotional regulation, social functioning, and engagement in personal projects. **Conclusions:** This reassignment of the voice from an external source to an internal one suggests that AT can modify how individuals relate to their voices and may empower them to regain control over their hallucinations. However, given the exploratory nature of this study, the results should be interpreted as examples.

## 1. Introduction and Clinical Significance

Auditory verbal hallucinations (AVHs) are commonly defined as a perception of verbal auditory (speech) that occurs in the absence of a corresponding external stimulus [1]. AVHs (also known as “voices”) are among the most prevalent symptoms of schizophrenia, affecting around 70% of this population [1,2]. How the voices manifest can vary greatly from one individual to another (e.g., loudness, linguistic properties, frequency, inter–outer space location, and content) [3,4,5,6,7]. Around 70% of voice hearers with schizophrenia attribute an identity to their voice, ranging from a vague idea of who the voice might represent to an unambiguous representation of who it might be [8,9]. Some may even assign their voice a name, a gender, an age, and physical characteristics. The voice can, for example, represent someone close to the person (e.g., family member and neighbor), someone they have never seen, or a supernatural identity (e.g., demon) [8,10]. In this context, voice-hearers often establish personal relationships with their voices, sharing characteristics similar to real-life interpersonal relationships [4,5,11,12]. Patients also hold beliefs concerning their voices (e.g., omnipotence over the patient and malevolence), which may be influenced by the nature of that relationship [13,14]. These beliefs can also be related to the origin of the voice, as patients may attribute hearing voices to themselves (i.e., their own voice/thoughts) or to an external source (i.e., an external agent) [3,5,15]. In some instances, AVHs can have a devastating effect on a patient’s life, leading to feelings of depression and anxiety, impaired social functioning, high suicidal risk, and delayed recovery [2,16,17]. Indeed, many experience one or multiple voices that are perceived as intrusive, unintentional, unwanted, and distressing [5,18]. Unsurprisingly, the experienced level of distress appears to be related to the negative/derogatory content of voices (i.e., threatening, frightening, and hostile voices) and the patients’ beliefs about their voices [19,20,21,22].

Although pharmacological interventions have revolutionized the care of patients with schizophrenia and have allowed for symptomatic improvements, they have not been a cure that effectively addresses all areas affected by the illness (e.g., cognitive symptoms) and are associated with several side effects [23,24,25,26,27]. Moreover, approximately 30% of schizophrenia patients are resistant to pharmacological treatment, with most of them experiencing distress related to persistent AVHs [28,29,30]. Psychotherapy is also a promising avenue to improve the symptomatology and well-being of those living with schizophrenia, and it can be a helpful adjunct to antipsychotic medication as well. For AVHs specifically, a variant of cognitive behavioral therapy for psychosis (CBTp) has been developed with the aim of challenging and modifying beliefs about voices using Socratic questioning, behavioral experiments, and cognitive restructuring [31]. This approach allows patients to change their behaviors related to psychotic symptoms, reduces distress, and improves patients’ functioning and well-being [32,33,34]. CBTp has shown numerous benefits in reducing the core symptoms of schizophrenia, and its use in combination with pharmacotherapy is usually recommended in those with treatment-resistant schizophrenia. However, up to 50% of these patients fail to improve in a clinically significant manner [35,36,37]. Thus, therapeutic developments have encouraged patients to dialog with their voices [38]. Additionally, integrating other therapeutic methods has helped highlight voice-hearing aspects, such as the relationships between voice hearers and their voices [11,39]. Indeed, dialogical psychotherapeutic interventions explicitly encourage patients to engage with their voices and embrace the communicative aspect of the experience of voice hearing [40,41,42,43], which was hypothesized to be responsible for positive changes in the relationship between patients and their voices [11,41,42,44].

Particularly, Avatar Therapy (AT) is a novel intervention using dialogical components in a virtual environment to improve relationships with their voices and gain control over them. To do so, patients are first invited to create an avatar representing the voice that generates the most distress. This 3D character is then animated in real time by the therapist, who controls the voice, distances, and facial expressions. These interactions occur in a virtual environment, either on a computer screen or in virtual reality (VR), the latter requiring a specialized headset [41,42,45,46]. Across three randomized controlled trials by two independent teams, it was consistently shown that AT could significantly decrease AVHs to a greater extent than treatment-as-usual (d = 0.52, *p* = 0.009), supportive counseling (d = 0.80, *p* = 0.009), and CBTp (d = 1.08, *p* < 0.001) [41,42,47].

Throughout the immersive sessions, the avatar commonly uses confrontational (e.g., provocation) and positive techniques (e.g., questioning self-perceptions or beliefs and empathetic listening) [48,49]. Notably, questions about the patient’s beliefs (i.e., origin of the voice, intentions of the voice, and mental illness) or discussions enabling the patient to reconsider their beliefs generally occurred around the fourth to sixth session. For example, the avatar can suggest that the patient seems to influence their voice or that the AVHs could perhaps be self-generated and reflect what the patient thinks of himself [48]. In response, patients often reconsidered their beliefs regarding their voices as well as their self-perceptions, using progressively more and more self-appraisal and self-affirmation as therapy progressed [48].

While our research team has been practicing this approach for over a decade, it has also been described by another independent team that initially developed this intervention [49]. Indeed, another qualitative study aimed at identifying the therapeutic targets of AT described that the therapist (through the avatar) worked toward an internal attribution for understanding the voice (i.e., reflecting a part of the self) in 14 of the 53 participants [50]. Moreover, it was previously observed that one patient was able to reattribute the voice as a part of himself and later yielded an excellent therapeutic response [49]. Considering this, it is possible that this reassignment of the voice from believing that the source is external to oneself could be one of the mechanisms by which AT can modify the ways of relating with voice and allow the patient to gain control over their AVHs. Finally, a similar phenomenon of drastic self-attribution of the voices was observed in three participants selected from a previously published clinical trial evaluating the effectiveness of virtual reality-assisted AT [41]. Particularly, these participants all decided to change the identity of their avatar to represent themselves, thereby engaging in a dialog with themselves. The objective of this study is to explore the therapeutic mechanisms involved in patients who engaged with the propositions made by the avatar and modified their beliefs about the origin of the voices. In an attempt to document and gain understanding of this understudied phenomenon, this case series aims to explore (1) the evolution of the reattribution of the participants’ most distressing voice to oneself during the course of AT and (2) the patients’ perception of this reattribution.

## 2. Case Presentation

### 2.1. Participants

Three participants with treatment-resistant schizophrenia were explicitly selected in this case series on the basis that they fully modified their beliefs about the origin of their most distressing voice during the course of AT, ranging from an external to a self-attributed origin. They were all part of a larger randomized trial evaluating the efficacy of Avatar Therapy (Clinicaltrials.gov identifier number: NCT03585127) [41]. Of the 37 participants who completed TA in this trial, the therapy evolved for three of them in such a way that their avatar became a representation of themselves. They were also the only three patients to experience such a shift in this previous trial [41]. The trial was conducted in accordance with the Declaration of Helsinki and was approved by the institutional ethical committee (CER IPPM 16-17-06). Written informed consent from all patients was obtained. Participants were referred by their treatment team, and recruitment occurred at the Institut Universitaire en Santé Mentale de Montréal (Montreal, QC, Canada). To be included in the trial, they had to be at least 18 years old, have a DSM-IV diagnosis of either schizophrenia or schizoaffective disorder (confirmed using the French version of the Structured Clinical Interview for DSM-IV), live with persistent and distressing AVHs, and be resistant to pharmacological treatment (i.e., failure of two or more antipsychotic trials). Participants were excluded if they presented a neurological disorder, a substance use disorder, underwent CBTp in the past year, or had an unstable/serious physical illness. During the entire duration of this study, participants continued to receive their standard psychiatric care, although their medication dosage remained stable during therapy.

### 2.2. Avatar Therapy

**Figure 1:**
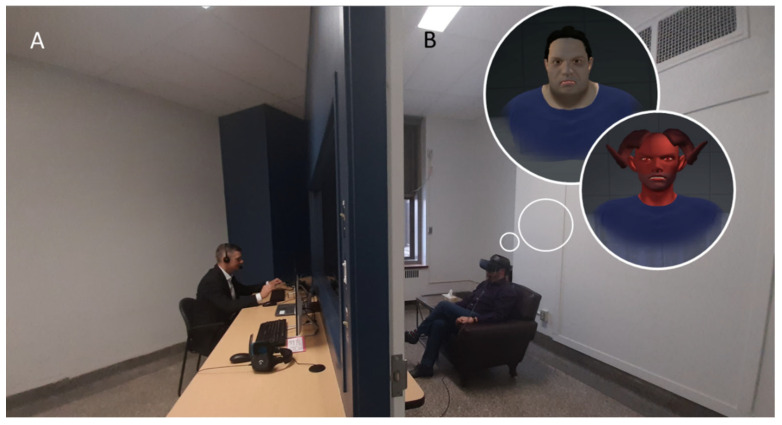
Setup of the immersive sessions. (**A**) Therapist’s side and (**B**) participant’s side. The participant, wearing a VR headset and noise-canceling headphones, sat in an adjacent room and was invited to enter a dialog with their avatar in the VR environment. The therapist could see the participant through a one-way mirror.

Patients completed nine one-hour sessions of Avatar Therapy delivered by an experienced psychiatrist (AD) with over 10 years of experience working with this complex population. During the first session, participants were invited to create an avatar representing their most distressing voice, which was selected based on the patient’s self-report. This first step was accomplished in VR immersion, which was facilitated by a head-mounted display, and they personalized their avatar’s physical appearance as they imagined the voice to look and the characteristic of the voice (e.g., pitch, volume) to match what they heard as closely as possible (Figure 1). As homework for the next session, the patient was asked to note all the content of the week’s voices without censorship. During the second session, the therapist recreated the voice’s typical discourse, using examples of what the patient had provided. Then, throughout the following sessions, the therapist specifically targeted emotional regulation, self-perception (i.e., self-esteem), and coping skills by progressively modulating the avatars’ speech and tone to resonate with the patients’ newfound assertiveness. This intervention aims to change the relationship between the avatar and the patient, evolving from confrontational, controlling, and powerful to a constructive and more positive dialog [48]. Each therapeutic session (session 2 and onward) consists of the following three parts: (1) pre-immersion: summary of the preceding week and determination of the objective(s) of this therapy session between the patient and the therapist; (2) immersion (5–20 min): patient dialogs with an avatar, which is animated by a live voice of the therapist, while trying different adaptation strategies in real-time; and (3) post-immersion: debriefing of the participant’s experience, including the feelings that arose during the immersion and goals for the next week. For the second part of each session, participants experienced VR immersion using an Oculus Rift head-mounted display. The VR environment was created using a custom-made Unity 3D game engine, featuring unique avatars created via the Morph3D Character System. Lip-syncing was achieved with the SALSA with Random Eyes Unity 3D extension, while a Roland AIRA VT-3 voice transformer modified the avatar’s voice in real time. Facial expressions were programmed to convey emotions based on the Facial Action Coding System. The setup material and the therapy itself were described in more detail elsewhere [41,46]. A specificity of this intervention is that it is highly customizable; the therapist can adjust to the patient’s speech in real time during immersion. For example, although a scenario may have been discussed beforehand in pre-immersion, it may evolve or diverge during immersion, leading the therapist to adapt the dialog and therapeutic strategies accordingly. Moreover, the avatar–patient relationship develops following each participant’s therapeutic progression. Participants are also encouraged to set their own objectives for the upcoming session. Therefore, although it was not routinely performed or suggested, changing the avatar’s name or appearance was possible upon the patient’s request. In the present study, the therapist had to adapt to these three cases, notably by helping them to cope with their new understanding of their voices. Always according to the patient’s perception, the therapist who initially embodied the distressing voice has, following the reattribution of the voice to the self, embodied a part of the patient. The relationship thus shifted to represent a more concrete interaction between different parts of the patient’s self.

### 2.3. Measures and Analysis

The evolution of the self-appropriation of the voice was documented based on the audio recordings of each immersive session (second to ninth session). Each of the three participants completed eight immersive sessions, resulting in a total of 24 immersive sessions (385 min), along with one post-therapy semi-structured interview each (totaling 275 min). Immersive sessions and interviews were audio-recorded and transcribed by S.G. Following multiple reviews of the audio recordings and transcripts, S.G. produced narrative summaries incorporating direct quotations from the participants. All authors cross-validated the interpretation of the participant quotations to ensure consistency and accuracy. Specifically, dialogs about the patient’s beliefs regarding the voices and the avatar’s questions aimed at encouraging reflection on the topic were selected to illustrate the evolution of the self-attribution of voice. Moreover, semi-structured interviews were conducted by a research nurse to gather more details about the participants’ perceptions of their experience undergoing AT. Structured interviews were analyzed using content analysis to extract thematic categories related to life changes following Avatar Therapy, common across all three participants. This thematic analysis was performed by L.D. and validated for consistency by S.G. The presented verbatims were translated from French to English by L.D. and M.B. In addition, the severity of AVHs was assessed by a trained research psychiatric nurse before, one week after therapy, and at 3-month follow-up after the end of the therapy using the total auditory hallucination subscale score of the Psychotic Symptoms Rating Scale–Auditory Hallucinations Subscale (PSYRATS-AH) [51]. The questionnaire comprises 11 items evaluated by interview, with each item ranging from 0 to 4 (total score ranging from 0 to 44), and has shown excellent interrater reliability and good validity [51]. In addition to the total score of this questionnaire, specific items of interest have been examined, including the following:

- Beliefs about the origin of the voice, ranging from (0), meaning no voices are present, and (1), believing voices to be solely internally generated and related to oneself, to (4), believing the voices are solely due to external causes (100% conviction).
- Intensity of the distress associated with hearing voice(s), ranging from (0), meaning that the voices are not distressing at all, to (4), meaning the voices are extremely distressing.

## 3. Results

### Case Illustration of Voice Reattribution

**Case 1—Mr. A**

At the time of enrollment in this study, Mr. A was a man in his late thirties who had been diagnosed with schizophrenia 11 years prior. Despite trials with first-generation antipsychotics (e.g., haloperidol, clopixol, and loxapine), second-generation antipsychotics (e.g., paliperidone), as well as clozapine and multiple courses of electroconvulsive therapy, Mr. A. remained symptomatic. He heard numerous voices at a time, which he identified as “human souls in pain”. The initial avatar represented a hostile, offensive, and denigrating woman whom he had known in the past. From the second session, Mr. A started to assert himself and faced the avatar with conviction and confidence. Thus, during the third session, the therapist (through his incarnation of the avatar) was already able to suggest that the voice could be originating from himself. Mr. A was initially somewhat ambivalent about this idea, as shown in the following verbatim:

*Avatar: We are together in your brain; I am a part of you.*

*Mr. A: Yes, you could be a part of me, that I don’t know. But I don’t like you.*

The same was true during the fourth session, during which the avatar continued to suggest that the voice reflects what he thinks of himself.

*Mr. A: What would be good would be for me to try to be a good person, and for you to create a future with me. Instead of you dragging me down or always trying to make me anxious. I don’t see the effect it has. I wonder why you do it, and how you do it.*

*Avatar: I am not doing anything; you are capable of doing it all on your own.*

In the pre-immersive face-to-face discussion with the therapist at the beginning of the fifth session, Mr. A mentioned that the content of the voices had changed, and he now attributed them to the other residents where he lived. For example, the voices could criticize him for smoking too much. When questioned about the meaning of those changes, Mr. A stated that this could have occurred because he did not feel good about his decisions regarding his lifestyle. From that moment, the therapist and Mr. A mutually decided that the avatar would represent a part of himself moving forward. Therefore, Mr. A decided to rename the avatar after his own name and mentioned that he now wanted their relationship to change.

*Avatar: I wish we could get along now. You always think I’m picking on you.*

*Mr. A: You’re like a part of me, deep down, so if we could remolecule together* [forming a whole with the parts of himself] *and form…*

During the sixth session, Mr. A. suggested creating a new avatar with the patient’s physical characteristics.

*Mr. A: Yes, you look like the old avatar I created; you don’t have a choice.*

*Avatar: I always have a choice. Would you like me to change?*

*Mr. A: Yes, maybe at some point, we’ll change you. We’ll make you like me.*

Finally, for the seventh session, the avatar’s physical appearance was changed to resemble Mr. A’s. During this session and the following ones, the avatar represented the patient’s pessimistic thoughts. During these last sessions, acceptance of the illness and oneself was addressed to help Mr. A reconcile with the part of himself that he had difficulty accepting.

*Mr. A: We will no longer be victims. I am no longer a victim; you are not a victim. We must accept ourselves as we are. We must find friends who will respect us and love us as we are. Saying this to you is challenging. We must accept that we have committed criminal acts.*

For Mr. A, AVH severity decreased from a score of 27 at baseline to 2 after therapy, representing a 92.6% reduction. At 3-month follow-up, AVH severity returned to almost the same level as the baseline, with a score of 26. Mr. A, who was 100% convinced that the voices were produced solely by an external voice source, changed his belief about the origin of the voice. Indeed, after therapy, he had no voices, and at the 3-month follow-up, Mr. A was 50% or more convinced that the voices came from an external source. While the voices are extremely distressing and the distress was the worst you can have at the baseline, they are no longer distressing at all after therapy. Nevertheless, at the 3-month follow-up, the intensity of distress related to AVHs was back to being very distressing.

Despite this, in the qualitative interview conducted 5 months after the end of therapy, Mr. A. attributed this shift in his beliefs to developing better self-perceptions. In addition, he mentioned that this reappropriation of the voice allowed a reduction in distress related to hearing voices.

*Mr. A: It seems like my little personal voice is getting louder and louder, and I perceive more love from this voice, which feels good.*

In summary, throughout the sessions, Mr. A showed a progression from an external to an internal attribution of the origin of the voice. This led to the creation of a new avatar, named after himself, representing his pessimistic thoughts. In the final sessions, Mr. A reconciled with this avatar, who represents a part of himself that he had previously struggled to accept. After the therapy, as well as at the 3- and 5-month follow-ups, the degree of conviction regarding the origin of the voices appeared to fluctuate over time along a continuum. However, when Mr. A believed that the voices originated from himself, he associated this shift with reduced distress, a more positive relationship with the voices, and an improved self-perception.

**Case 2—Ms. B.**

Ms. B. was a woman in her late fifties with a complex set of spiritual beliefs. She was diagnosed with schizoaffective disorder about 30 years before the present study. At pre-therapy, she was receiving treatment with aripiprazole. In the first session, Ms. B created an avatar representing a supernatural entity that was condescending and insulting towards her, in addition to mandating her to commit suicide. During the second session, Ms. B spontaneously responded by asserting herself and using self-affirmation, highlighting her strengths/qualities to contradict the avatar’s insults. Thus, during the third session and onwards, the avatar started suggesting that the voice could come from her. Ms. B quickly agreed by explicitly expressing that the avatar reflected her.

*Avatar: I know you hate yourself.*

*Ms. B: A little.*

*Avatar: A lot. Why do you think I’m telling you that all the time?*

*Ms. B: Because I’m the one who tells myself that unconsciously. I know it, you’re my mirror.*

*Avatar: How do you want us to reconcile if you hate me? I’m inside you, I know it. You hate me to death.*

The reattribution of the avatar gradually took place throughout therapy. Indeed, during the fourth session, Ms. B expressed that there could be a link between her self-perception and the relationship between her avatar and herself.

*Avatar: So, being with me is fun, now.*

*Ms. B: More and more, but not all the time. But when things are not going well, it’s me, not you. I understood that.*

*Avatar: What exactly did you understand?*

*Ms. B: I understood that you are a part of me that is inseparable, and that everything I experience in relation to you is what I am worth deep down inside my being. From there, if I understand how to modify certain aspects of my attitude, it will change the whole relationship more and more between the two of us, and we will become good friends.*

During the fifth and sixth sessions, the relationship between the avatar and Ms. B became more positive. Ms. B started recognizing that a positive part of the voice could be a part of herself. She also spontaneously mentioned that she was thinking about changing the name of her avatar to represent this change in her beliefs. During the seventh session, Ms. B decided to formally change the name of the avatar to her own without changing the physical appearance of the avatar. At that time, she saw a positive future for their relationship and expressed the wish to accept this part of herself.

*Avatar: You changed my name.*

*Ms. B: It’s because I consider you to be me. If we keep a name that is outside of me, it will not be a part of me. If I want us to continue on the same line, if I call you* [name of the avatar], *it doesn’t click. If I call you “Beautiful* [Ms. B]” *it improves, it amplifies, it illuminates who you are and, for me, it enters an infinitely more beautiful, more luminous energy. You have already been an entity, you have already been many things, but now, I don’t see that in you anymore. I see that you are a part of me that wants to improve, that we move forward and grow together.*

During the last two sessions, Ms. B made peace with the part of herself that she had devalued for most of her life.

*Avatar: Are you freeing yourself from me?*

*Ms. B: Yes, I am freeing myself from the part of me that has hurt me my whole life, which has been heavy to bear, which has asked a lot of me, and which has saddened me.* […] *For me, we have walked together, we have experienced beautiful things, but what I’m feeling now is that it’s me who’s speaking to myself. Whether you have this appearance changes absolutely nothing. It’s me who’s speaking to myself. You are a part of me.*

After the end of the therapy, the severity of Ms. B’s AVHs decreased by 15.6% (from a score of 32 to 27) and continued to decline at 3-month follow-up (score of 22). Concerning the beliefs about the origin, Ms. B was initially more than 50% convinced that the voices came from an external source. After the therapy, she believed that the voices were uniquely produced and related to herself, which was maintained at the 3-month follow-up. However, the intensity of the distress associated with the voices remained the same directly after therapy before decreasing at the 3-month follow-up (from very to moderately distressing).

At the time of her qualitative interview, 6 months after the end of therapy, the reattribution of the voice to oneself was maintained. Ms. B. notably mentioned that the content of the voices had evolved positively. In addition, she again verbalized that the voices were directly associated with her self-perceptions.

*Ms. B: I felt that it was changing. It was no longer the same words. I see it differently. It still annoys me, but I realized that it is a part of me that is simply talking to itself. It’s my unconscious that is talking to me. It’s not someone else. This voice does not come from elsewhere; it comes from me.* […] *It’s the disease, because this voice is my unconscious that is talking to me, because I have underestimated myself too much all my life. I have sabotaged myself too much. I have seen myself as less than nothing, so it gave the color of the voice that corresponds today to what I hear.*

In summary, in Ms. B’s case, a link emerged throughout the sessions between her self-perception and the evolving relationship with her avatar. She began to recognize that a positive aspect of the voice could reflect a part of herself and expressed a desire to accept it. The avatar came to represent a part of herself that she had devalued for most of her life and with which she reconciled. At post-therapy, as well as at the 3- and 6-month follow-ups, the reattribution of the voice was maintained, and she reported that the content of the voices was directly related to her self-perception.

**Case 3—Mr. C.**

Mr. C. was a man in his late thirties diagnosed with schizophrenia for about a decade. Mr. C remained symptomatic despite pharmacological trials with first-generation antipsychotics (e.g., pimozide) and second-generation antipsychotics (e.g., olanzapine and risperidone). Although he was abstinent at the time of enrollment in this study, the content of his voices was mainly related to his substance use history. His avatar represented the voice of a drug dealer he had known in his past, who encouraged him to use drugs and demanded that he pay off debts from drug purchases. High levels of distress were present, primarily associated with the fear of relapsing. During the second and third sessions, Mr. C. tried to assert himself by responding to the avatar’s comments but had difficulties doing so. During the pre-immersive discussion of the fourth session, the therapist introduced the idea that the avatar could represent a part of himself and might be relevant to discuss with it. At this point, Mr. C answered that, although this could be true, he was not feeling ready. During the fifth session, the avatar continued to occasionally suggest that he and the voice were the same entity. Mr. C then stated that the voice was an evil part of himself and that he perceived himself as a victim. During this session, the beginning of a discourse of acceptance emerged.

*Mr. C: I’m tired of hearing your little demon voice. If it’s part of me, I’ll get used to it, and I’ll be able to get the upper hand on it.*

Furthermore, during this session, Mr. C addressed his avatar using his own name. From this moment on, he began to consider that this part of himself could also become positive.

*Mr. C: Crack is strong, I can’t touch that anymore. Don’t touch that,* [Mr. C].

*Avatar: Did you just change my name?*

*Mr. C: Yes. If I want, I’ll call you* [Mr. C] *now.*

*Avatar: Ah! So I’m not a devil anymore?*

*Mr. C: No, you have a good side,* [Mr. C].

During the sixth and seventh sessions, Mr. C changed the avatar’s identity so that he was now representing the reunion of the part of himself that he did not like and the one that he loved. The dialog included elements about accepting past situations that were making him feel ashamed. Spontaneously, Mr. C expressed his desire to change their relationship. During that session, Mr. C and his avatar discussed how they would build a better relationship to consolidate a future together.

*Mr. C: I told you I don’t need you anymore… No, I want to be with you, but I want us to live in harmony.*

*Avatar: How are we going to do that?*

*Mr. C: I have plans. By carrying out my plans one step at a time, at some point, you may not show up for me anymore.*

At the beginning of the eighth session, the avatar’s physical appearance was changed to include physical characteristics similar to Mr. C’s. This session’s focus was the reconciliation between the part of him that wanted to use drugs and the part of him that wanted to move forward and set up life projects without drugs.

After therapy, the severity of Mr. C’s AVHs slightly decreased, from a score of 25 to 24. Regarding his beliefs about the origin of the voices, Ms. B was initially more than 50% convinced that the voices came from an external source. After the therapy, he believed that the voices were uniquely produced and related to himself. The intensity of voice-related distress also decreased from very distressing to moderately distressing.

During the qualitative interview, one month after the end of therapy, in addition to maintaining the reattribution of the voice to oneself, Mr. C noted that his own negative self-perceptions caused the content of the negative voice.

Until the 3-month follow-up, the severity of AVHs continues to decrease (score of 14). The belief that the voices were uniquely produced and related to him was maintained, but the voices went back to being very distressing.

*Mr. C* *: With Avatar Therapy, I know it comes from me.*

*Research nurse: In what way?*

*Mr. C* *: I think it’s to harm me. I’m hurting myself.*

*Research nurse: Why?*

*Mr. C* *: Well, because I don’t like myself.*

When asked by the evaluator how he concluded that the voice was coming from himself, Mr. C mentioned that he noticed the content of the voice evolved based on his self-esteem levels. Additionally, taking ownership of the voice brought him a sense of calm and empowerment.

*Mr. C* *: It’s because I was starting to love myself more. I told myself, if I’m the one talking to myself, it’s going to have a more effective impact.*

*Research nurse: How did you start loving yourself?*

*Mr. C* *: When I realized that I was the one who was putting these voices in myself, that I was able to control them, it was like a relief.*

In summary, Mr. C’s beliefs about the origin of the voice evolved throughout therapy as he viewed the voice as an evil part of himself and perceived himself as a victim who needed to accept it. He created a new avatar to represent a reunion between the part of himself he disliked and the part he valued. After therapy, Mr. B maintained the belief that the voices were entirely self-generated, a view that remained stable at the 1- and 3-month follow-ups. Mr. C. associates the content of the voice with his level of self-esteem. Attributing the voice to himself gave him a sense of control over the voice and resulted in him feeling better.

**Main changes reported after AT**

During the interview after the therapy, three main themes emerged among the 3 participants: change in emotional and social aspects as well as activities and projects (Table 1).

## 4. Discussion

This case series highlights the experience of three individuals with treatment-resistant schizophrenia or schizoaffective disorder who, during AT, progressively identified changes in their beliefs about the origin of their AVHs. This process led to the recognition that voices, which were initially perceived as having an external origin, were actually internally generated thoughts, reflecting how they perceived themselves. This realization led all three participants to change the identity of their avatar; two of them even asked to recreate an avatar that resembled them better. In addition, the severity of the AVHs improved for two out of three participants. Interestingly, participants mentioned that their belief about the origin of their voices positively impacted their self-perceptions and how they related to their voices. Beyond the voices, improvements were also observed in several areas, including emotions, social aspects, and project development. The current section will discuss the possible underlying mechanisms of AT enabling this drastic change and the potential impacts of this self-attribution.

Firstly, AT specifically aims to improve the patients’ self-perception, notably by encouraging the participants to be assertive and use self-valuation while responding to confrontation by the avatar. As their self-perception evolved, the patients’ sense of self may be seen as becoming unified as it became clear to them that they were discussing with themselves. Moreover, improvements in self-perception can lead to improved symptoms and reduced AVH severity [52]. Indeed, this aligns with studies showing that self-perceptions and beliefs about the voices are interrelated [53,54,55]. Moreover, many voice-hearers have negative self-perception, which is reflected in the content of persecutory voices and the perception that the voice is omnipotent [49,53]. Indeed, for these three cases, self-views and past experiences were integrated in the voices’ negative content. Because of the association with their past experiences and insecurities, this phenomenon often makes voice-hearers particularly vulnerable to the derogatory and threatening content of voices [39]. Changing dysfunctional core beliefs/schemas and increasing the activation of positive adaptive beliefs could be deemed important. Therefore, improved self-perception may have an impact on the content of voices. In this case, these changes could have made the patients realize that their self-perception influences the content of voices and, therefore, that the voice originates from themselves.

Secondly, changes could also emerge from the shift in dialog that occurs during AT, where the avatar gradually begins to question the patients’ belief systems. At the same time, the content becomes less threatening and more supportive. By enabling patients to engage with their personified voice, with an emphasis on altering their emotional experience and targeting elements they desire to work on, this intervention may allow changes in distorted beliefs about themselves, their voices, and the future [56,57]. AT differs from other psychotherapeutic interventions in that it introduces a third person in the therapeutic relationship: the avatar. Therefore, the immersion taking place during AT could be described as a trialogue. Although immersive sessions mainly consist of a conversation between the patient and the avatar, the therapist also acts as a regulator by regularly interrupting the interaction and intervening as himself to make suggestions, encourage the patient to adopt specific strategies, or even suggest ending the immersion session. The use of the avatar allows the introduction of ideas through a “familiar” character, thus enabling the therapist to bring up subjects that would typically be difficult to broach in such a short course of psychotherapy with a therapist the participants had never met before. For example, questioning the patient’s beliefs about the origin of their voice might be more accepted, less confrontational, and lead to more changes when performed through an avatar. This is a much more direct approach than that used in traditional psychotherapies (e.g., CBTp), which focus primarily on changing the way the voices are cognitively perceived [58].

Finally, these participants could represent a specific subset of patients undergoing AT (i.e., three of the 37 participants in the AT group of the randomized trial) that would be more susceptible to such a drastic change in beliefs. Indeed, all three showed self-assertiveness earlier in the course of AT than what has been previously described in a larger sample [48]. Moreover, belief in the origin of voices is a continuum ranging from the conviction that they come from an external source to the conviction that they come from oneself. A complete reattribution of voice leading to the creation of a new avatar representing a part of the patient’s self was relatively rare in our sample (8.1%). Intermediate beliefs between these two extremes were observed in the previous trial, with a significant change in the attribution of voice of the subscales of PSYRATS-AH from baseline to 3-month follow-up (d = 0.665, *p* = 0.004). It would be interesting to analyze session by session (as opposed to only administering the questionnaire before and after treatment) in a future study, using a scale such as PSYRAT or using a visual Likert-type rating scale with a rating system. Moreover, they could have greater cognitive flexibility about their insight into their illness and experiences than other patients with treatment-resistant schizophrenia. These characteristics could have enabled them to change their perspectives and to believe that their voices were internally generated and related to their self-perceptions. Thus, some participants with poorer coping strategies, self-esteem, or flexibility may need more sessions to accept that the voice could be coming from them.

Although multiple hypotheses were formulated, further studies will be needed to determine how these changes occur and whether this self-attribution positively impacts AT’s effectiveness. Thus far, this therapeutic phenomenon appears to be a promising avenue, as all three cases appear to have subjectively benefited from the therapy in some way. Nevertheless, it remains unknown whether using an avatar and immersive virtual reality is necessary to bring change toward voices becoming unified with the patient or whether such results might occur with other standard dialogical treatments. Nevertheless, this change appears to be linked to the realization, through AT, that there was a clear link between their self-perception and the origin of the voices, which could represent a key therapeutic mechanism of AT. This intervention is distinct in that it allows the content of the avatar to change throughout the sessions and is more confrontational, leading to stronger emotions than what may be expected with other relational interventions. Therefore, further studies comparing dialogical interventions are warranted. While improvements in these participants’ lives were observed, the reattribution aspect does not appear to be necessary to obtain an interesting therapeutic response [41]. In addition, the level of conviction in beliefs about the voices does not precisely follow the severity and distress associated with the voice, suggesting that reattribution alone may not be sufficient as a sustained mechanism for symptom relief. A forthcoming study is planned to identify therapeutic mechanisms and predictors of positive treatment response. Thus, this study was exploratory; therefore, the results should be interpreted as examples. In the future, further analyses will be conducted to assess whether this phenomenon is linked to treatment response in participants who underwent a large-sample, single-blind, randomized controlled trial comparing AT with CBTp that is currently underway.

## 5. Conclusions

In conclusion, this case series represents a tentative attempt at describing a novel phenomenon that has been observed during AT: the reattribution of the voices during therapy. This paper is of interest as solely a limited number of studies have explored how relationships with voices and beliefs about the voice change over time during psychotherapeutic interventions (i.e., [49,59,60]). Results shown in this paper support AT as a promising tailored method that allows voices and related beliefs to evolve in treatment-resistant patients. As shown, these patients were able to drastically change their beliefs through the course of AT by attributing their voices to an internal origin, meaning that they recognized that their voices were self-generated. Through this process, two patients asked to create a new avatar that represented them instead of an external character, thereby reuniting the voice with themselves. Future studies should continue to investigate the association between changes in beliefs about the origin of the voice and the evolution of treatment-resistant patients. Moreover, analysis of whether a particular patient profile would be favorable to reattributing the voice should be the subject of a future study.

## Figures and Tables

**Table 1:** Qualitative improvements observed in all three participants after Avatar Therapy.

	Mr. A	Ms. B	Mr. C
Emotional changes	*“I am less sad.”*	*“I find myself wonderful, and I adore myself more and more intensely.”*	*“Today, I love myself.”*
Social changes	*“I am maybe more comfortable with people. […] I am less in my bubble, less isolated.”*	*“My good friend told me: you are becoming really zen, you are not the same [Ms. B] I knew before.”*	*“My brother, we see each other three to four times a week, but we talk every day. […] Before the therapy, he wouldn’t even answer when I called, and I couldn’t see the children, my sister-in-law didn’t want me to…”*
Projects and activities	*“Short-term, it is to spend my summer having fun as much as possible and get in shape, and then this fall it is to go to the carpentry activity and continue art.”*	*“I did a show as a singer; I’d been hearing voices for 33 years and waiting to do it.”* *“I want to do a benefit concert.”*	*“After therapy, I discovered yoga. And that, that really helps me a lot.”*

## Data Availability

The original contributions presented in this study are included in the article. Further inquiries can be directed to the corresponding author.
